# Gpbar1 agonism promotes a Pgc-1α-dependent browning of white adipose tissue and energy expenditure and reverses diet-induced steatohepatitis in mice

**DOI:** 10.1038/s41598-017-13102-y

**Published:** 2017-10-20

**Authors:** Adriana Carino, Sabrina Cipriani, Silvia Marchianò, Michele Biagioli, Paolo Scarpelli, Angela Zampella, Maria Chiara Monti, Stefano Fiorucci

**Affiliations:** 1 University of Perugia, Department Surgical and Biomedical Sciences, Perugia, Italy; 2 University of Perugia, Department of Medicine, Perugia, Italy; 3 University of Naples Federico II, Department of Pharmacy, Naples, Italy; 4 University of Salerno, Department of Pharmacy, Fisciano, Salerno, Italy; 5 University of Perugia, Department of Experimental Medicine, Perugia, Italy

## Abstract

Gpbar1 is a bile acid activated receptor for secondary bile acids. Here we have investigated the mechanistic role of Gpbar1 in the regulation of adipose tissues functionality in a murine model of steatohepatitis (NASH). Feeding wild type and Gpbar1^−/−^ mice with a high fat diet-fructose (HFD-F) lead to development of NASH-like features. Treating HFD-F mice with 6β-ethyl-3a,7b-dihydroxy-5b-cholan-24-ol (BAR501), a selective Gpbar1-ligand, reversed insulin resistance and histologic features of NASH, increased the weight of epWAT and BAT functionality and promoted energy expenditure and the browning of epWAT as assessed by measuring expression of Ucp1 and Pgc-1α. The beneficial effects of BAR501 were lost in Gpbar1^−/−^ mice. *In vitro*, BAR501 promoted the browning of 3T3-L1 cells a pre-adipocyte cell line and recruitment of CREB to the promoter of Pgc-1α. In conclusion, Gpbar1 agonism ameliorates liver histology in a rodent model of NASH and promotes the browning of white adipose tissue.

## Introduction

Non-alcoholic fatty liver disease (NAFLD) is the most common cause of chronic liver disease in the Western countries and a leading cause of liver-related morbidity and mortality worldwide^1–3^. NAFLD is broadly categorized into two phenotypes: non-alcoholic fatty liver (NAFL) which is characterized by isolated steatosis, and non-alcoholic steatohepatitis (NASH), which features hepatocytes ballooning, inflammatory cell infiltration and parenchimal fibrosis, that carry on a substantial risk to progress to cirrhosis and hepatocellular carcinoma, and is expected to surpass hepatitis C virus infection as the leading cause of liver transplantation, in the next decade^3^. Despite NASH represents a significant burden for public health systems worldwide, there are no approved drugs designed to treat this condition and, therefore, there is an urgent need to develop novel approaches specifically tailored to treat NASH patients.

Bile acids, the end products of cholesterol metabolism, are signaling molecules that activate both cell surface and nuclear receptors^4^. Gpbar1 (also known as TGR5 or MBAR) is a G protein-coupled receptor activated by secondary bile acids^5^, highly expressed in entero-hepatic tissues, pancreatic β cells, skeletal muscles and white and brown adipocytes (WAT and BAT). In these tissues Gpbar1 functions as a nutrient sensor by exerting genomic and non-genomic effects^4^. Non-genomic effects are mediated by changes in intracellular concentrations of cyclic-AMP (cAMP), leading to rapid phosphorylation of downstream kinases; while the recruitment of a phosphorylated cAMP-response element binding protein (pCREB) to a cAMP responsive element (CRE) in the promoter of target genes, mediates the transcriptional (genomic) effects of the receptor^6^. Gpbar1 is thought to regulate energy expenditure and glucose homeostasis^7^, but since Gpbar1^−/−^ mice had a normal span life and fail to develop overt metabolic abnormalities with age^8^, the role of the receptor remains poorly defined. Furthermore the liver parenchimal cells, hepatocytes^5^, lack Gpbar1, indicating that the effects exerted by this receptor on liver metabolism are indirect.

The 6β-ethyl-3a,7b-dihydroxy-5b-cholan-24-ol (BAR501), is a non-bile acid, steroidal Gpbar1 ligand^9,10^ that activates the receptor with an EC_50_ of ≈2 µM, in transactivation assay in transfected cells and that directly increases the release of glucagon-like peptide (GLP)-1 from intestinal endocrine cells^9^.

In the present study we have investigated the metabolic effect of BAR501 in a model of NASH, induced by feeding wild type and Gpbar1^−/−^ mice a high fat diet and fructose (HFD-F). In this model^11,12^, BAR501 effectively protected against liver damage by resetting the partition of lipid between liver and adipose tissues. Mechanistically we found that Gpbar1 activation increased the thermogenic functionality of BAT and promoted the browning of epididymal (ep)WAT as indicated by the increased expression of uncoupling protein Ucp1 (a protein found in the inner mitochondrial membrane), peroxisome proliferator-activated receptor (Ppar)- α and Pparγ coactivator 1 (Pgc-1)α, in the epWAT^12^. Furthermore, Gpbar1 activation by BAR501 in 3T3-L1 cells, a pre-adipocyte cell line, promoted the differentiation toward a brown phenotype as indicated by increased expression of Ucp1 and Pgc-1α. Finally, we provide evidence that regulation of Pgc-1α by BAR501 involves the binding of a CRE located in the promoter of this gene. Taken together, these results demonstrate that Gpbar1 agonism modulates WAT and BAT metabolism by genomic and non-genomic effects.

## Methods

### Chemicals

BAR501 (6β-ethyl-3a,7b-dihydroxy-5b-cholan-24-ol) was synthesized as described elsewhere^9^. The dose used in this study was chosen according to a previously published study^10^.

### Animal models

Twenty-four weeks old male Gpbar1^−/−^ mice^8,13,14^ on a C57BL6/Sj background and their congenic littermates were fed a high fat diet (HFD) containing 60% kj fat (ssniff ® EF acc. D12492 (I) mod.) and fructose (HFD-F) in drinking water (42 g/l) or normal chow diet (6 mice) for 18 weeks^12^. Food intake was estimated as the difference of weight between the offered and the remnant amount of food at 7-days intervals. The food was provided as pressed pellets and the residual spillage was not considered. After 9 weeks, HFD-F mice were randomized to receive HFD-F alone (9 mice) or in combination with BAR501, 15 mg/kg/day, by gavage (9 mice) for 9 weeks. Mice were housed under controlled temperature (22 °C) and photoperiods (12:12-hour light/dark cycle), allowed unrestricted access to standard mouse chow and tap water. The experimental protocol was approved by the Animal Care and Use Committee of the University of Perugia and by the Italian Minister of Health and Istituto Superiore di Sanità (Italy) and were in agreement with the European guidelines for use of experimental animals (permission n. 41/2014-B). The general health of the animals was monitored daily by the Veterinarian in the animal facility. At the day of sacrifice, fed mice were deeply anesthetized with a mixture of tiletamine hypochloride and zolazepam hypochloride/xylazine at a dose of 50/5 mg/Kg and sacrificed before 12 AM.

### Thermal images

BAT temperature was recorded through the study using a non-invasive technology. Briefly, mice were maintained at 25 °C and the thermal images were taken by a FLIR E6 thermal imaging camera (FLIR System, Wilsonville, Oregon) and the surface temperature quantified by the FLIR Tools^15^.

### Biochemical analyses, OGTT, ITT and bile acids assay

AST, triglyceride, total- and HDL-cholesterol and fasting glucose concentrations were quantified using an automated clinical chemistry analyzer (Cobas, Roche). Plasma insulin levels were measured by ELISA assay (Mercodia). The OGTT and ITT were performed after 9 and 18 weeks of HFD-F as described elsewhere^12^. Circulating bile acids were measured by a liquid chromatography-tandem mass spectrometry (MS/MS) analysis, as described elsewhere^12,13^.

### Histopathology

For histological examination, portions of the right and left liver lobes and epididymal adipose tissues were fixed in 10% formalin, embedded in paraffin, sectioned and stained with Sirius red and Hematoxylin/Eosin (H&E), for morphometric analysis. NASH severity (steatosis, hepatocytes ballooning, lobular inflammation and portal inflammation) was scored in H&E-stained cross sections using an adapted grading system of human NASH as described previously^12^. Hepatic fibrosis was evaluated in Sirius red stained sections has described previously^10^.

### Immunohistochemistry

Immunohistochemistry on epWAT and on BAT (used as positive control) were performed using formalin-fixed paraffin-embedded sections (7 μm thick) as described previously^12^. Sections were stained with an anti-Ucp1 (Abcam 1:500) antibody and counterstained with hematoxylin.

### Mice motor activity

Mice motor activity was monitored using Panlab Infrared (IR) Actimeter (Panlab Harvard Apparatus, Barcelona, Spain) and analyzed with V2.7 ActiTrack software system. Each animal motor activity was recorded for 10 minutes during the day every week. Data were shown as global motor activity i.e. sum of stereotypes and locomotion activity. Stereotyped movements during the analyzed interval, indicate the number of samples where the position of the mice is different from the position of the previous sample and equal to the position of the 2nd sample back in time. Locomotion activity indicates locomotion during the analyzed interval, i.e. distance walked in each sample where the position of the mice is different from the position of the previous sample and different of the position of the second sample back in time.

### Image analysis

Microscopy fluorescent images were acquired using AxioVision.Z1 microscope equipped with ApoTome filtering device and a AxioCam mRm digital camera (Carl Zeiss Microscopy). Captured fluorescent images of 3T3-L1 cultured cells were optimized with a factor 140, 6 px sharpening mask; images of liver sections were optimized with a factor 160, 8 px sharpening mask (Adobe Photoshop CC).

### Tissue microarray

Total RNA was extracted from liver, epWAT, BAT, gastrocnemius and terminal ileum (4 mice/group) using TRIzol reagent (Invitrogen) and reverse transcribed with SuperScript II Reverse Transcriptase (Invitrogen) following the manufacturer’s instructions. A total of 10 ng of cDNA was pipetted into each well of a 96-well Custom RT^2^ Profiler PCR Array [CAPM13349, RT^2^ Profiler PCR Array, Qiagen (http: www.qiagen.com/it/products/catalog/assay-technologies/real-time-pcr-and-Real-time PCR-reagents/rt2-qpcr-primer-assays/l)] and amplified following the manufacturer’s instructions in StepOnePlus instrument (Applied Biosystems). This RT^2^ PCR array was settled up to assess a total of 75 genes: 30 genes for liver, 6 for gastrocnemius, 6 for intestine, 22 for epWAT and 11 for BAT; 2 housekeeping genes were used for each tissue for data normalization. The RT^2^ Profiler PCR Array also includes control elements for: Genomic DNA contamination detection, RNA sample quality and General PCR performance. Data analysis is based on the ΔΔC_T_ method with normalization of the raw data to either housekeeping genes.

### Cell culture and cell differentiation

3T3-L1 preadipocytes were plated at 1.7 × 10^6^ in a T75 flask. Preadipocytes were grown to confluence in DMEM and 10% FBS [24]. Two days after reaching confluence (day 0), the cells were stimulated with differentiation mix (Mix) alone (DMEM, 10% FBS, 517 μM 3-isobutyl-1-methylxanthine, 1 μM dexamethasone, and 172 nM insulin). At day 2, Mix was replaced by insulin medium (DMEM, 10% FBS, and 172 nM insulin) alone. The medium containing insulin was replaced every 2 days. At day 8, cells were stimulated 18 hours with 10 or 50 μM BAR501. Then cells were treated according to the protocols established for the different experiments.

### Immunofluorescence

3T3-L1 cells at day 8 of differentiation were plated at 1 × 10^5^ on chamber slides (Nunc™ Lab-Tek™ II Chamber Slide™ System) for immunofluorescence and stimulated or not, with BAR501 (50 µM) 18 h. After the treatment cells were fixed in 4% PFA for 15 minutes at room temperature (RT) and then washed in PBS. After incubation with blocking solution (8% BSA, 0,1% Triton in PBS) 30 minutes, cells were incubated for 1 h RT with primary antibodies against UCP1 (Abcam 1:500 made in Rabbit). Then cells were first washed in PBS and then incubated with secondary antibodies Alexa Fluor 546 anti-Rabbit IgG (Molecular Probes, 1:300) for 45′ RT. Finally cells were washed and coverslips were mounted with SlowFade ®Gold Antifade Reagent with Dapi (Molecular Probes).

### Quantitative Real-Time PCR analysis

RNA extracted from cells (3T3-L1) and tissues (liver, epWAT, BAT, gastrocnemius and intestine) was subjected to reverse transcription. Real Time PCR: 10 ng cDNA were amplified in a 20 μL solution containing 200 nM of each primer and 10 μL of 2X SYBR FAST Universal ready mix (Invitrogen). All reactions were performed in triplicate, and the thermal cycling conditions were as follows: 10 min at 95 °C, followed by 40 cycles of 95 °C for 10 sec, 55 °C for 10 s and 60 °C for 30 sec in StepOnePlus instrument (Applied Biosystems). The relative mRNA expression was calculated and expressed as 2^−(ΔΔCt)^. Expression of the respective gene was normalized against B2M and GAPDH mRNA as an internal controls.

The following primers were used for Real-Time PCR : mouse-Gapdh: ctgagtatgtcgtggagtctac and gttggtggtgcaggatgcattg; mouse-B2m: ctttctggtgcttgtctcactg and ttcagcatttggatttcaatgt; mouse-Adiponectin: gacaggagatcttggaatgaca and gaatgggtacattgggaacagt; mouse-Srebp1c: gatcaaagaggagccagtgc and tagatggtggctgctgagtg; mouse-Pparγ: gccagtttcgatccgtagaa and aatccttggccctctgagat; mouse-Pparα: cagaggtccgattcttccac and gatcagcatcccgtctttgt; mouse-Fabp4: aaacaccgagatttccttcaaa and tcacgcctttcataacacattc; mouse-Ucp1: ctcactcaggattgtgcctctac and tctgaccttcacgacctctgta; mouse-Pgc-1α: cttagcactcagaaccatgcag and aatgctcttcgctttattgctc; mouse-Fgf21: acacagatgacgaccaagacac and aagtgaggcgatccatagagag; mouse-Cebpα: gacatcagcgcctacatcga and tcggctgtgctggaagag; mouse-Perlipin: gatcgcctctgaactgaagg and gcggcatattctgctgtctc; mouse-Leptin: gtgctggagacccctgtgtcgg and gcttggcggataccgactgcgtg; mouse-Col1α1: tgactggaagagcggagagt and agacggctgagtagggaaca; mouse-Prdm16: acggaagagcgtgagtacaaat and cgtgaacaccttgacacagttt; mouse-Cd137: ctccaagtacctctcccagcat and gttgtgggtagaggaacaaaac; mouse-αSma: tgtgctggactctggagatg and gaaggaatagccacgctcag; mouse-Tgfβ: ttgcttcagctccacagaga and tggttgtagagggcaaggac; mouse-Ntcp: ggtgccctacaaaggcatta and gttgcccacattgatgacag; mouse-Bsep: atgcttgtgaccctgcaaa and agatcgttgacggatggaag; mouse-Mrp4: gcaaagcccatgtaccatct and accacggctaacaactcacc; mouse-Mdr1: caggagcccattctctttga and cgatgaactggtggatgttg.

### Chromatin Immunoprecipitation

3T3-L1 cells at day 8 of differentiation were stimulated 24 h with 50 μM BAR501 or received the vehicle alone (1% DMSO). Chromatin immunoprecipitation (ChIP) assays were performed according the manufactured protocols (EZ-ChIP™ Upstate, #17–371). Briefly, cell extracts were sonicated and divided. Antibodies (5 µg) against pCREB and normal goat IgG were added for immunoprecipitation. The immunoprecipitated chromatin was recovered and purified. Pgc-1α promoter DNA was quantified by real-time PCR analysis using primers for the Proximal (for GGCCAAGTGTTTCCTTTTCTT, rev CAGCCTCCCTTCTCCTGTG) and Distal (for CCCTCTCCAAATTTTTGGTTC, rev CAACTCTTTTCAGCAAGTCATGT) Pgc-1α promoter. Primers spanning a region located ~ 95.000 bp downstream of the Pgc-1α transcriptional start site were used as control (for CCCGGTATCTTGAGTTGTGTT, rev AGCAGGGTCAAAATCGTCTG). At least three replicates of each group were performed.

### Statistical analysis

All of the data are shown as the means ± SEM. Difference among groups was estimated using one-way ANOVA followed by Tukey’s post hoc test or by the T-test analysis when appropriated (GraphPad Prism 5.0 software). Significance was set up at *p* < 0.05.

## Results

### Effects of HFD-F on wild type and Gpbar1^−/−^ mice

**Figure 1:**
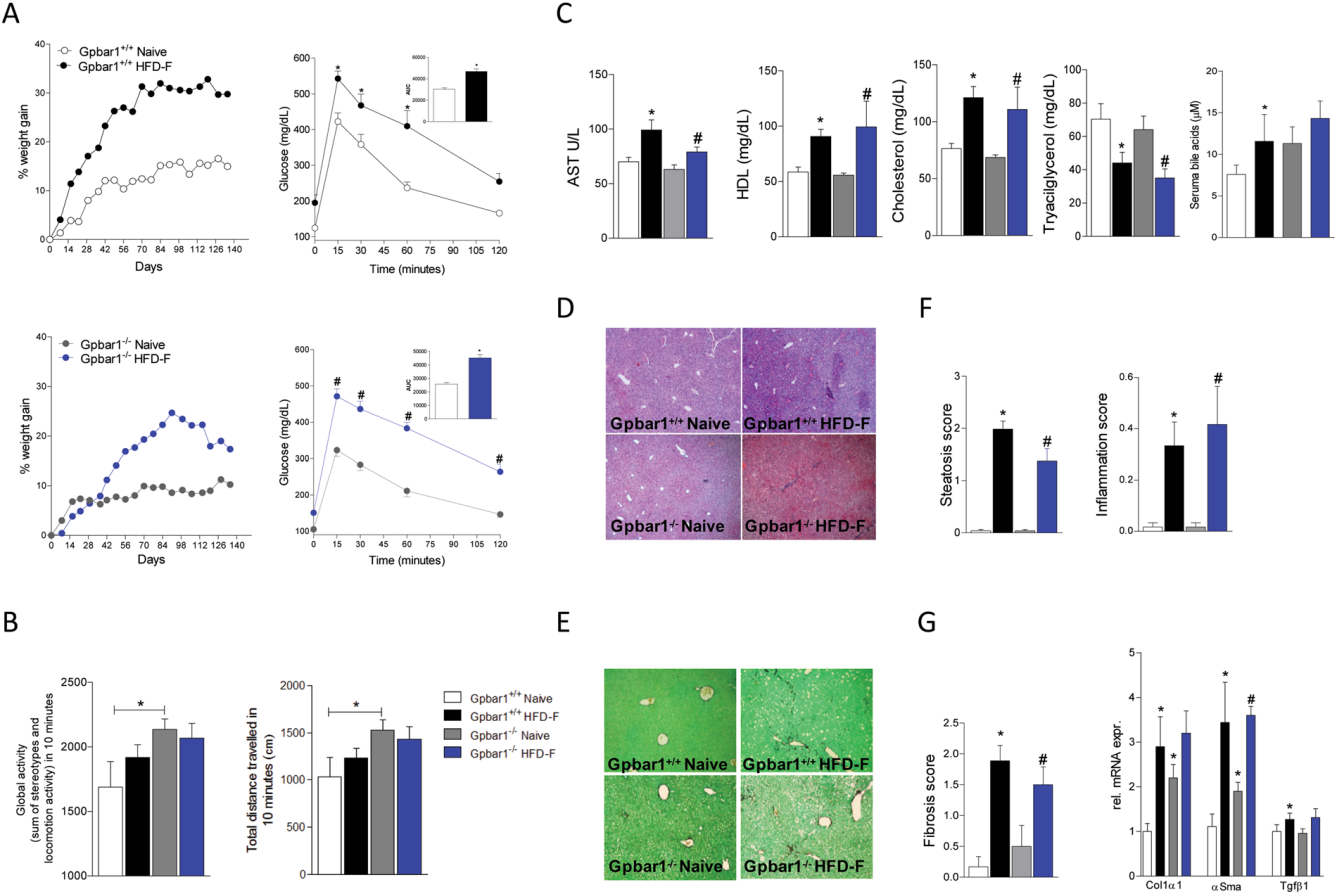
Gpbar1^+/+^ and Gpbar1^−/−^ mice feed HFD-F for 18 weeks develop a similar metabolic phenotype, insulin resistance and NASH-like phenotype. Wild type and Gpbar1^−/−^ mice were fed a HFD-F for 18 weeks. The data shown are: (**A**) Body weight (% delta weight) and glucose plasma levels response to oral glucose tolerance test (OGTT) carried out after 18 weeks of HFD-F; (**B**) Motor activity (Global activity and Total distance); (**C**) Plasma levels of AST, cholesterol, HDL, triacylglycerols and serum Bile Acids measured at the end of the study. (**D**–**F**) Liver histopathology obtained after feeding mice a HFD-F diet for 18 weeks. Liver sections where stained with Hematoxylin and eosin (**H** and **E**) staining (**D**), or (**E**) Sirius red. (**F**) Steatosis score and Inflammation score; (**G**) Fibrosis score and liver mRNA expression of pro-fibrotic genes. Values are normalized to β2-Microglobulin and Actin-β, the relative mRNA expression is expressed as 2^(−ΔΔCt)^ as described in Materials and Methods. Results are the mean ± SE of 5–9 mice per group. *p < 0.05 versus Gpbar1^+/+^ naïve mice, ^#^p < 0.05 versus Gpbar1^−/−^ naïve mice.

Feeding with a HFD-F for 18 weeks resulted in a ≈30% increase of body weight in both wild type and Gpbar1^−/−^ mice. However, Gpbar1^−/−^ mice gained weight slower than their congenic littermates with a statistically significant difference detected up to week 4 (Fig. 1A, p < 0.05; n = 8–10). No significant difference in body weight was detected in the 6–14 week period, but, Gpbar1^−/−^ were unable to maintain the body weight despite an equal decline of food intake was recorded in both mice strains (not shown). Assessing physical activity of both mice strains throughout the study revealed that, in comparison to wild type mice, Gpbar1^−/−^ had significantly higher physical activity (Fig. 1B), which might explain why Gpbar1^−/−^ under HFD-F gained body weight slower than their congenic littermates^14^.

**Figure 2:**
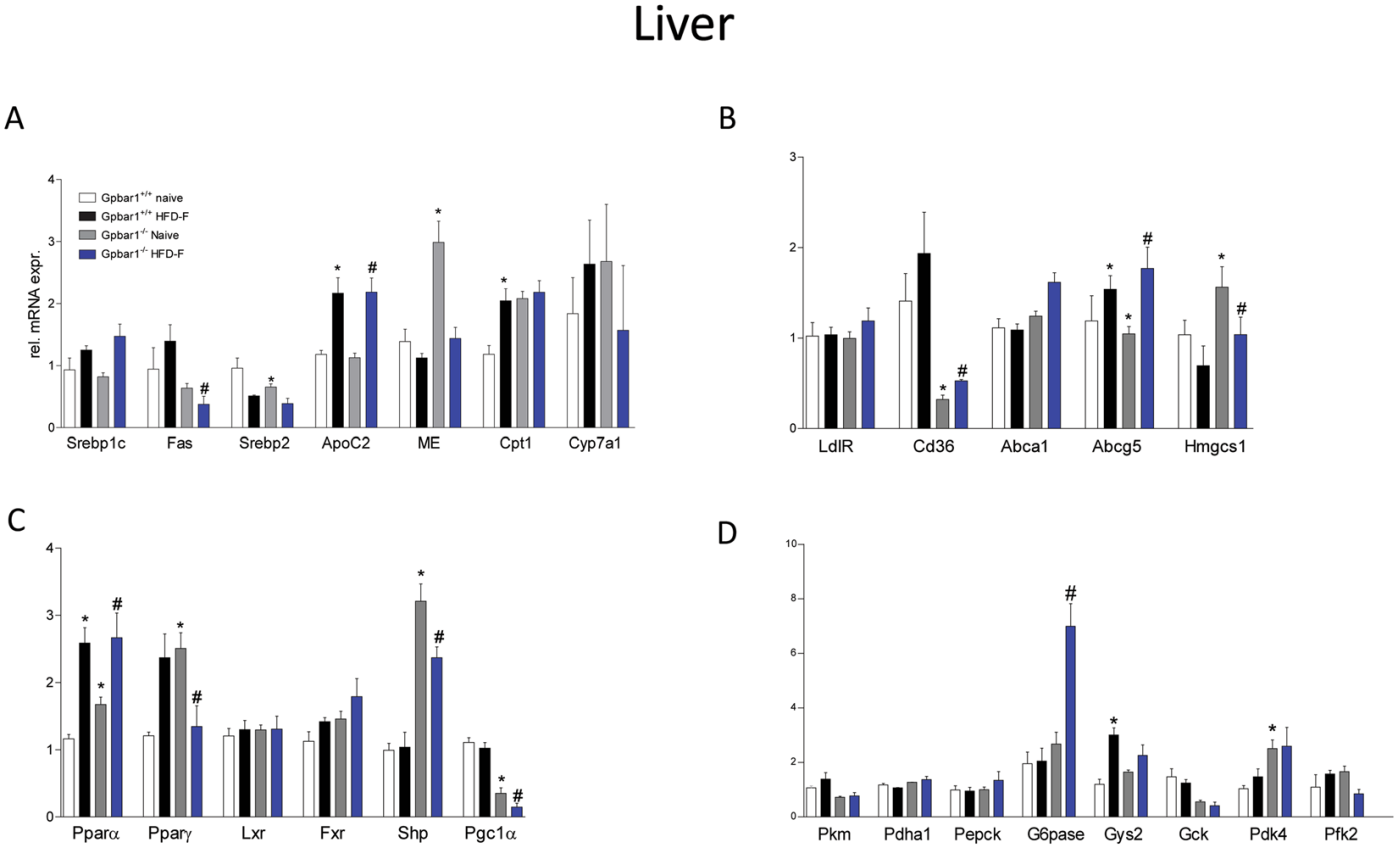
Feeding Gpbar1^+/+^ and Gpbar1^−/−^ mice with a HFD-F diet for 18 weeks results in a similar liver metabolic phenotype. Hepatic expression of genes involved in: (**A**) Triglycerides and fatty acids metabolism; (**B**) Cholesterol metabolism; (**C**) Nuclear receptors; (**D**) Glucose metabolism. Data are the mean ± SE of 5 mice per group. *p < 0.05 versus Gpbar1^+/+^ naïve mice, ^#^p < 0.05 versus Gpbar1^−/−^ naïve mice. Values are normalized to β2-Microglobulin and Actin-β, the relative mRNA expression is expressed as 2^(−ΔΔCt)^ as described in Materials and Methods.

At the end of the study, wild type and Gpbar1^−/−^ mice on HFD-F, were insulin resistant, as shown by results of OGTT (Fig. 1A) and ITT (not shown) and developed the same blood lipid profile than their congenic littermates (Fig. 1C; P < 0.05; n = 8–10). The histopathology analysis of livers after 18 weeks of HFD-F demonstrated appearance of NASH-like features in both wild type and Gpbar1^−/−^ mice, and no difference was detected among the two groups in terms of liver weight (not shown) and severity of liver steatosis, as measured by assessing ballooning, inflammatory, fibrosis scores and liver expression of pro-fibrotic marker genes (Fig. 1D–G; P < 0.05). However, the gene array analyses carried out on liver, epWAT, BAT, muscles (gastrocnemius) and terminal ileum, revealed that exposure to HFD-F regulates the expression of several genes in Gpbar1-dependent manner (Fig. 2). The liver expression of Fas, Cd36 and Pgc-1α was significantly reduced in Gpbar1^−/−^ naïve mice compared to wild type mice, while expression of malic enzyme (ME), hydroxymethil glutaryl CoA (Hmgs)1, Pparγ and Shp was increased. No changes were detected in the liver expression of other genes involved in lipid metabolism (Fig. 2A–C; P < 0.05). Feeding a HDF-F resulted in differential regulation of the liver expression of several genes such as ApoC2, Pparα and Pparγ, that were increased in wild type mice but not in Gpbar1^−/−^ mice (Fig. 2A–C; P < 0.05).

Few changes were also detected in the liver expression of glucogenetic and glycolytic genes. However, Gpbar1^−/−^ mice demonstrated a robust upregulation of G6Pase when fed a HFD-F (Fig. 2D; P < 0.05).

**Figure 3:**
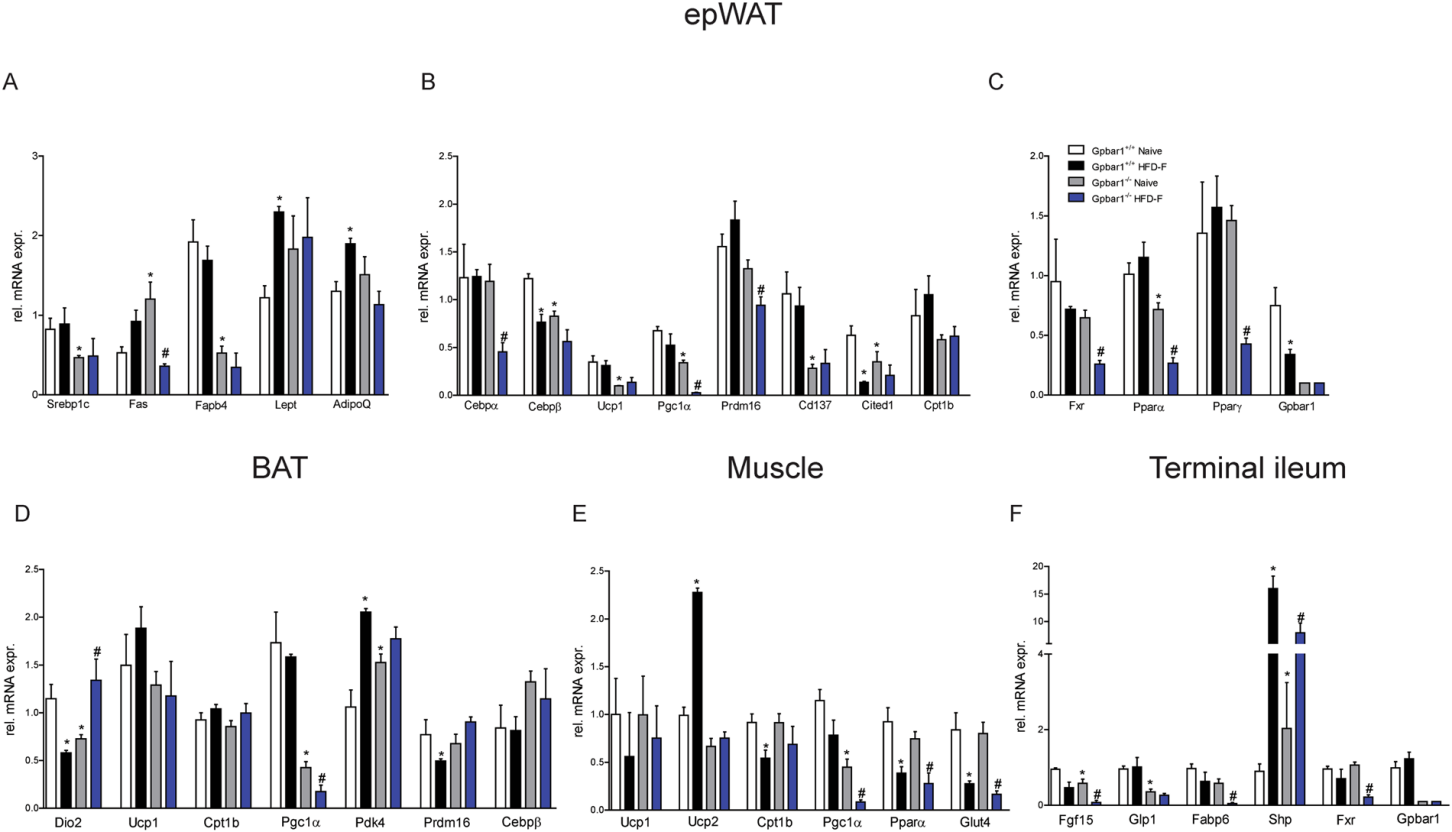
Feeding a HFD-F differentially modulates adipose tissues, intestinal and muscle metabolism in Gpbar1^+/+^ and Gpbar1^−/−^ mice. Panels A-C: mRNA expression of epWAT genes. Data shown are relative mRNA expression of genes involved in: (**A**) Adipogenesis and fatty acids transport and metabolism; (**B**) Brite/beige transdifferentiation; and (**C**) Nuclear receptors. Panels D–F: Effects of HFD administration on mRNA expression in the: (**D**) BAT, (**E**) Muscle (gastrocnemius), and (**F**) Terminal ileum. Data are the mean ± SE of 5 mice per group. *p < 0.05 versus Gpbar1^+/+^ naïve mice, ^#^p < 0.05 versus Gpbar1^−/−^ naïve mice. Values are normalized to β2-Microglobulin and Actin-β, the relative mRNA expression is expressed as 2^(−ΔΔCt)^ as described in Materials and Methods.

In comparison to wild type mice on a chow diet, naïve Gpbar1^−/−^ mice had a reduced expression of Ucp1, Pgc-1α, Cd137, Cited1, Srepb1c, Fabp4 mRNA in the epWAT (Fig. 3A–C, P < 0.05). Exposure of Gpbar1^−/−^ mice to the HFD-F resulted in a further downregulation of the expression of Cepbα and β, Ucp1, Pgc-1α, Fas and Fabp4 (Fig. 3A,B), and Fxr, Pparα and Pparγ, (Fig. 3C; p < 0.05) in the epWAT.

The widespread reduction of Pgc-1α mRNA in mice harboring a disrupted Gpbar1 was confirmed by analysis of BAT (Fig. 3D), muscle (along with Ucp2) and terminal ileum (Fig. 3E, F). Further on, Gpbar1 gene ablation resulted in a profound reduction of the intestinal expression of Fgf15 and Glp-1 (Fig. 3F). Feeding Gpbar1^−/−^ mice with a HFD-F further impaired the intestinal expression of Fgf15 and Glp1 along with Fabp6 and Fxr (Fig. 3F; P < 0.05). Taken together these results demonstrate that, in comparison to wild type mice, the liver expression of the large majority of metabolic genes is maintained in Gpbar1^−/−^ mice fed a HFD-F. However, because Gpbar1^−/−^ mice show an enhanced physical activity, it is unclear whether some of the observed changes are truly dependent on gene deletion or represent and adaptation to increased energy demand or both.

### BAR501, a Gpbar1 ligand, protects against development of NASH

**Figure 4:**
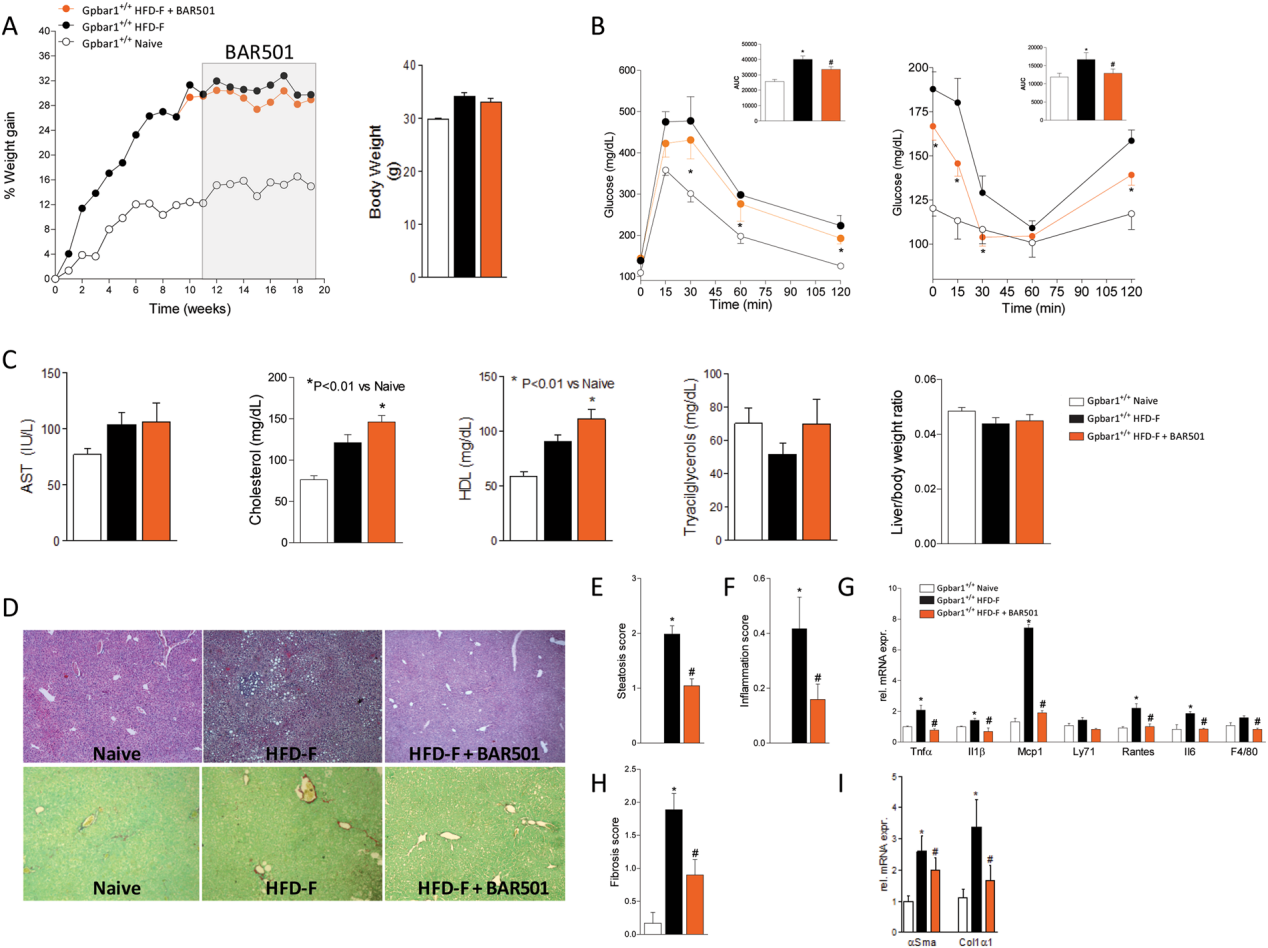
Treating wild type mice with a Gpbar1 ligand, BAR501, reverses NASH like features and redirect lipid partition in mice fed HFD-F. BAR501, 15 mg/kg/day, was administered by gavage starting on day 63 (week 9) for additional 9 weeks. The data shown are: (**A**) body weight (% weight gain and grams); (**B**) Glucose plasma levels response to oral glucose tolerance test (OGTT) and to insulin-tolerance test (ITT) at the 18^th^ week of HFD-F. (**C**) Plasma levels of AST, cholesterol, HDL and triacylglycerols measured at the end of the study; liver body weight ratio. The data shown in Panels A–C, are mean ± SE of 9 mice. (**D**) Hematoxylin and eosin (**H** and **E**) staining and Sirius Red staining of liver tissues obtained at the end of the study (19 weeks of HFD-F). The data are mean ± SE of 9 mice. Panels E–I. Effects of BAR501 on: (**E**) Steatosis (steatosis score); (**F**) inflammation (inflammation score) and (**G**) hepatic expression of pro-inflammatory genes; (**H**) Fibrosis score and (**I**) hepatic expression of αSMA and Col1α1 mRNA. Data are the mean ± SE of 5–9 mice per group. *p < 0.05 versus Gpbar1^+/+^ naïve mice, ^#^p < 0.05 versus Gpbar1^+/+^ HFD-F mice. Values are normalized to β2-Microglobulin and Actin-β, the relative mRNA expression is expressed as 2^(−ΔΔCt)^ as described in Materials and Methods.

We have then investigated whether BAR501, a selective Gpbar1 agonist, rescues wild type mice from NASH-like features caused by feeding a HFD-F. After 9 weeks, mice on a HFD-F gained significantly more weight than naïve mice (≈30% versus 10%, *P* < 0.01) (Fig. 4A) and become insulin resistant as demonstrated by results of OGTT and ITT performed at this time point (data not shown). These over-fed, insulin-resistant, mice were randomized to continue on the HFD-F alone or to be treated with BAR501 (15 mg/kg/day) for further period of 9 weeks. At the time of sacrifice, mice on a HFD-F alone, had a slight increase in plasma levels of AST and cholesterol while triglyceride levels were unchanged (Fig. 4C). At necroscopy, HFD-F mice showed NASH-like features with diffuse hepatocytes ballooning and microvescicular steatosis (Fig. 4D,E; P < 0.05). Analysis of inflammatory scores along with expression of inflammatory markers (Tnfα, Il-1β, Mcp-1, Rantes, Il-6) (Fig. 4F,G) demonstrated that feeding a HFD-F causes liver inflammation and an increase of inflammatory cells (F4/80) (P < 0.05 versus naïve mice). Additionally, the HFD-F caused a significant fibrosis as demonstrated by analysis of the extent of collagen surface in liver sections stained by Sirius red and analysis of expression of fibrosis markers, αSma and Col1α1 mRNA (Fig. 4H,I; P < 0.05).

Treating HFD-F mice with BAR501 had no effect on body weight (not it increased the physical activity, data not shown) but the Gpbar1 ligand improved insulin sensitivity as assessed by OGTT and IGTT carried out at the end of the study (Fig. 4B; P < 0.05). A statistically significant difference was detected at each time point from 30–120 min at the OGTT and at each time point (with exception of 60 min) at the ITT (Fig. 4B). Additionally, exposure to BAR501, increased plasma cholesterol and HDL levels (Fig. 4C).

Treating mice with BAR501 improved liver histopathology leading to a 50–70% reduction in the steatosis, fibrosis and inflammatory scores, along with reduction of inflammatory (Il-1β, Il-6, Tnfα, Mcp-1, Rantes and F4/80) and fibrogenic (αSma and Col1α1) genes (Fig. 4E–I; P < 0.05).

**Figure 5:**
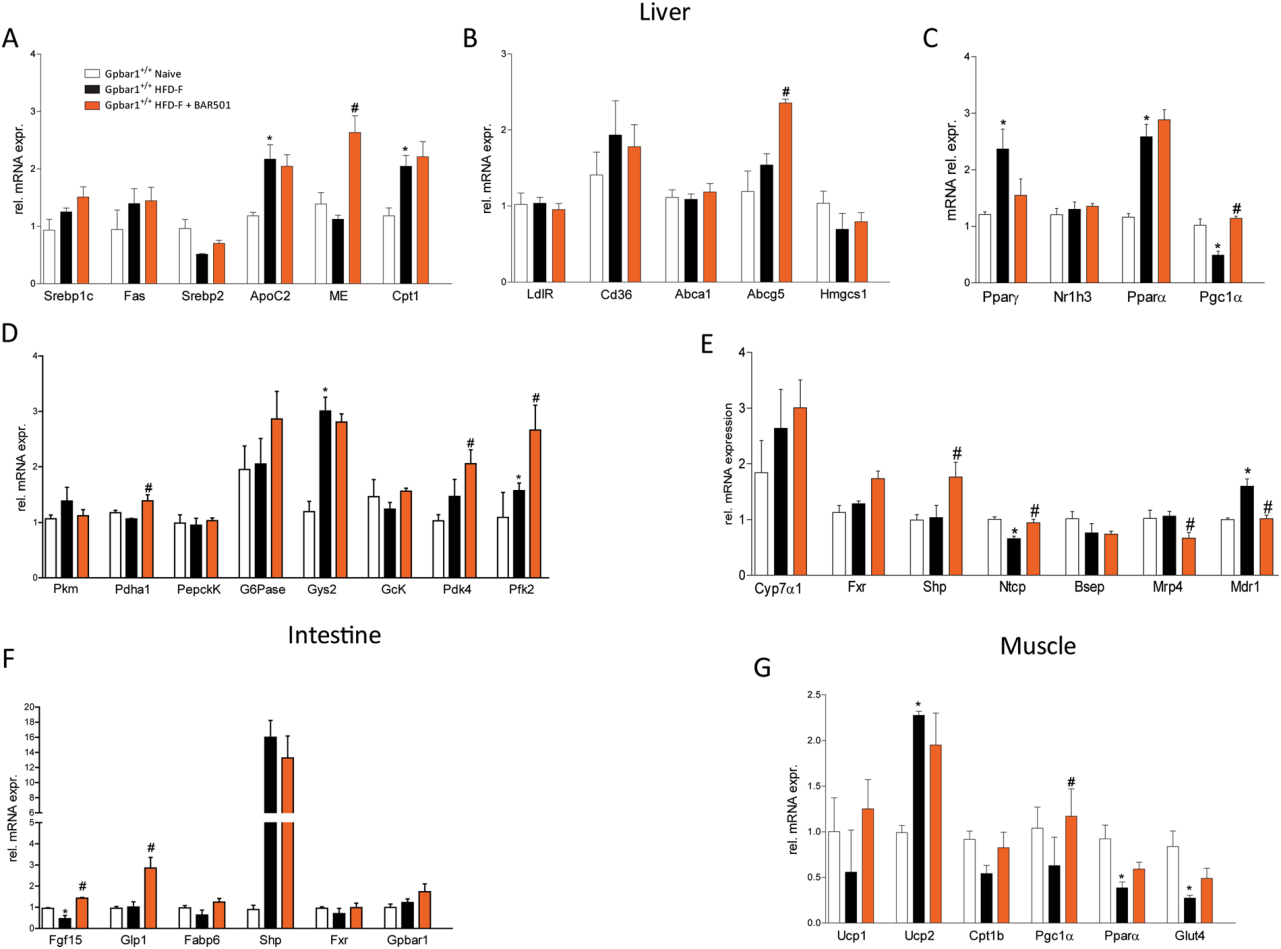
Effects of BAR501 on liver, muscle and intestinal metabolism. Change in transcript levels of genes involved in: (**A**) Liver triacylglycerols and fatty acids metabolism, (**B**) Liver cholesterol metabolism, (**C**) Liver nuclear receptors, (**D**) Liver glucose metabolism and (**E**) Liver Bile Acids transport and metabolism. Change in transcript levels of genes regulating: (**F**) Intestinal metabolism; and (**G**) Muscular metabolism. Data are the mean ± SE of 5 mice per group. *p < 0.05 versus Gpbar1^+/+^ naïve mice, ^#^p < 0.05 versus Gpbar1^+/+^ HFD-F mice. Values are normalized to β2-Microglobulin and Actin-β, the relative mRNA expression is expressed as 2^(−ΔΔCt)^ as described in Materials and Methods.

BAR501 increased the liver expression of malic enzyme (ME), Abgc5 (Fig. 5A and B) and Pgc-1α (Fig. 5C) and negatively regulated the expression of Pparγ (Fig. 5C), and modulated the expression of Pdk4 and Pfk2 (Fig. 5D; P < 0.05), and of genes involved in bile acids metabolism and transport such as Shp, Ntcp, Mrp4 and Mdr1 (Fig. 5E; P < 0.05). Finally, treatment with BAR501 also increased the expression of Fgf15 and Glp1 in the terminal ileum (Fig. 5F; P < 0.05) and Ucp1 and Pgc-1α in the muscle (Fig. 5G; P < 0.05).

### BAR501 induces browning of the epWAT

**Figure 6:**
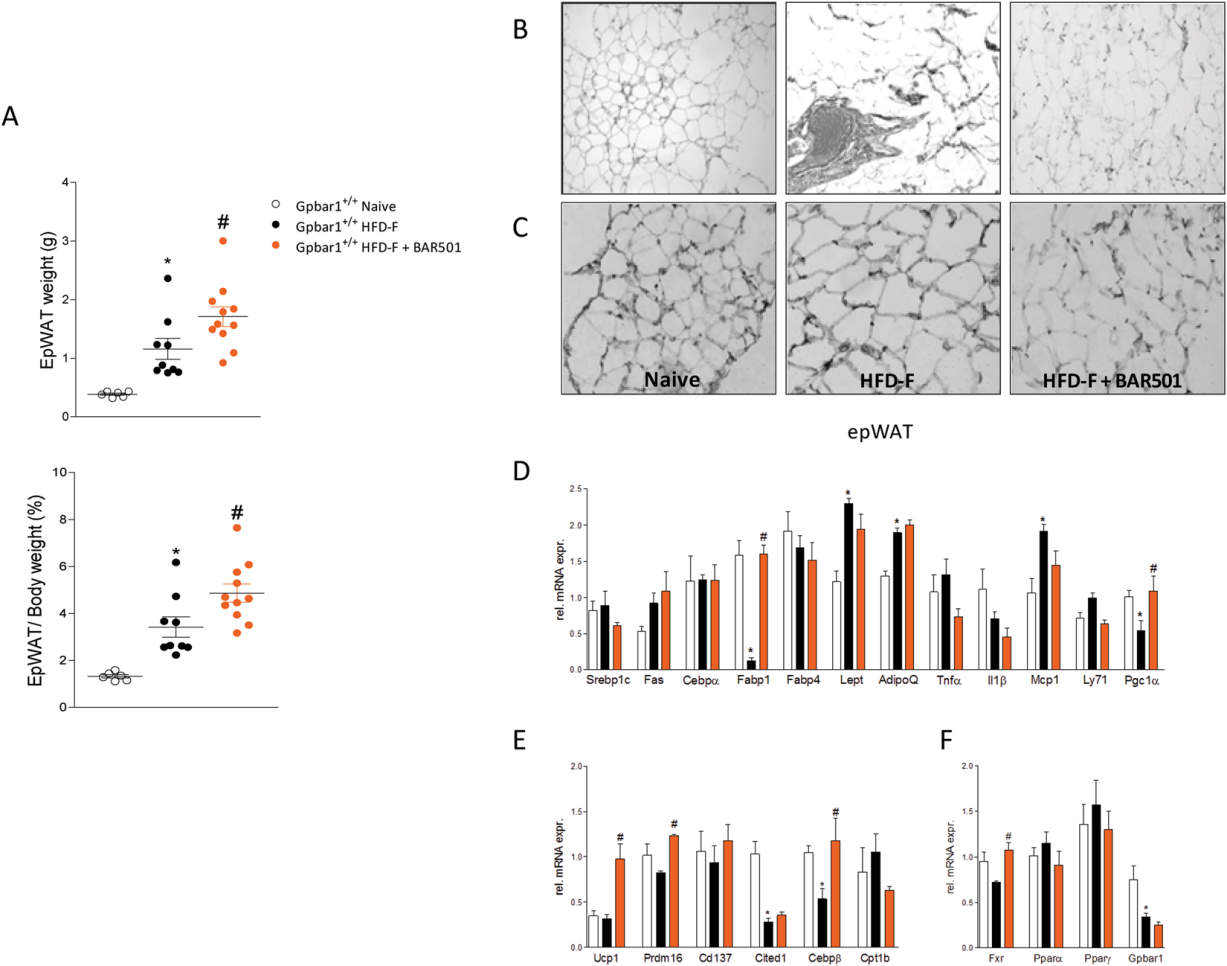
Gpbar1 agonism by BAR501 promotes the browning of epWAT. (**A**) epWAT weight (mg) and epWAT/Body weight ratio (%) obtained from experimental groups after 19^th^ weeks of feeding with a HFD-F alone or in combination with BAR501, 15 mg/kg/day. *p < 0.05 versus Gpbar1^+/+^ naïve mice, # p < 0.05 versus Gpbar1^+/+^HFD-F, n = 6–11 mice. (**B**) H&E staining on epWAT. Magnification is 20X; (**C**) Immunohistochemistry analysis of UCP1 protein in epWAT. Magnification is 20x. (**D**) Effects of BAR501 on mRNA expression of epWAT genes: (**D**) Adipogenesis and fatty acids transport and metabolism; (**E**) Brite/beige transdifferentiation; and (**F**) Nuclear receptors. Results are the mean ± SE of 5 mice per group. *p < 0.05 versus Gpbar1^+/+^ naïve mice, ^#^p < 0.05 versus Gpbar1^+/+^HFD-F mice. Values are normalized to β2-Microglobulin and Actin-β, the relative mRNA expression is expressed as 2^(−ΔΔCt)^ as described in Materials and Methods.

Feeding mice with a HFD-F for 18 weeks increased the weight of epWAT by ≈3 folds (P < 0.01) (Fig. 6A). The over-nutrition also resulted in changes of the epWAT morphology with appearance of large adipocytes along with an influx of F4/80 inflammatory macrophages (Fig. 6B). Dosing mice with BAR501 caused a further increase in epWAT weight (Fig. 6A; P < 0.05) and promoted its browning as assessed by measuring the expression of Fabp1, Pgc-1α (Fig. 6D; P < 0.05) and Ucp1, Real-time PCR and immunohistochemistry (Fig. 6C and E; P < 0.05). BAR501 also modulated the expression of other brite marker genes as Prdm16 and Cebpβ (Fig. 6E; P < 0.05), and slightly impacted on Nuclear receptors expression (Fig. 6F; P < 0.05).

### BAR501 increases the weight and thermogenic activity of BAT

**Figure 7:**
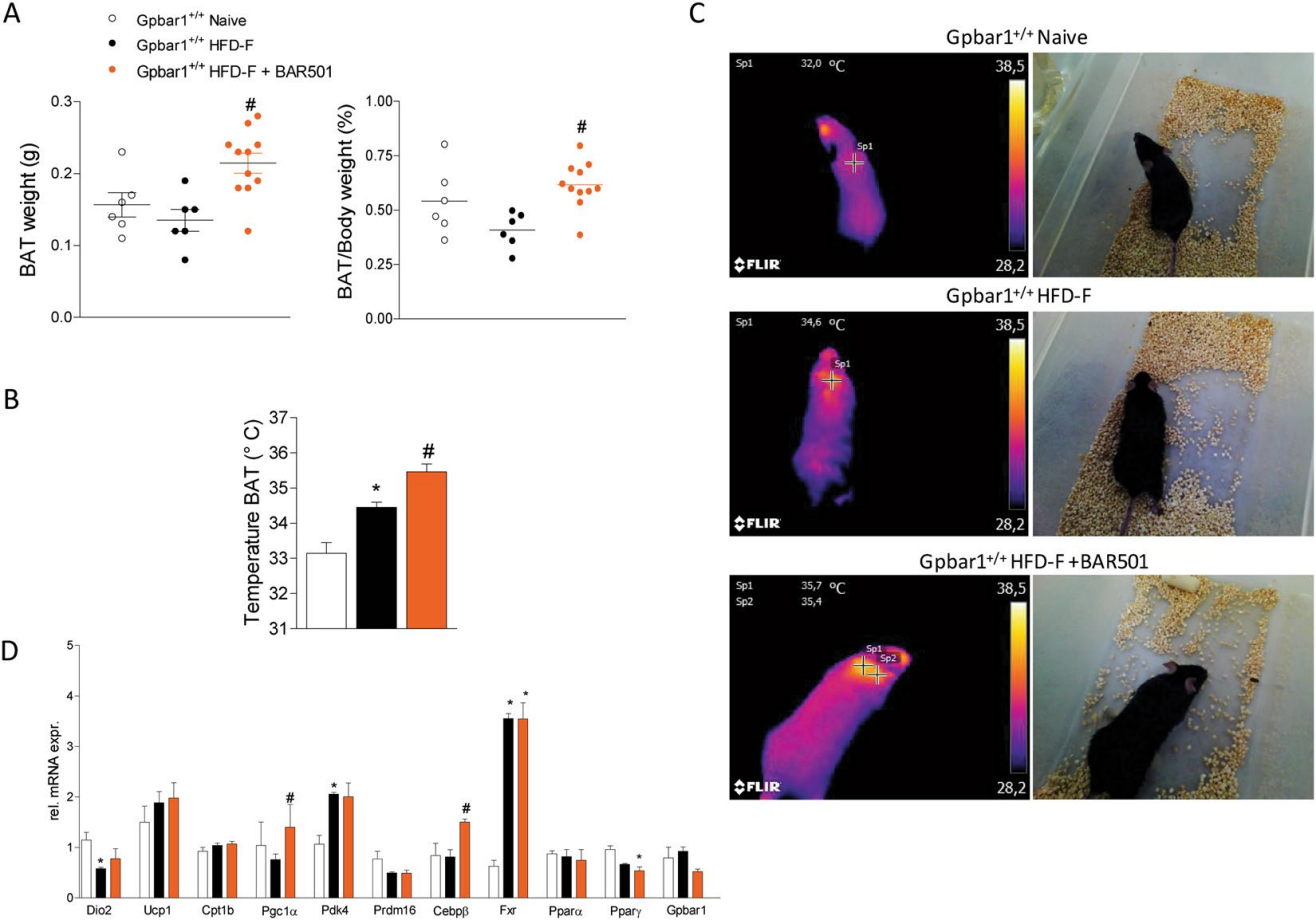
Gpbar1 agonism by BAR501 increases the weight and improves the functionality of BAT. (**A**) BAT weight (mg) and BAT/Body weight ratio (%) obtained from experimental groups after 19 weeks of feeding with a HFD-F alone or in combination with BAR501, 15 mg/kg/day. # < 0.05 versus Gpbar1^+/+^HFD-F, n = 6–11 mice. (**B** and **C**) BAT temperature measured by infrared images of interscapular area after 8 weeks treatment with BAR501. Data are mean ± SE of 6–11 mice per group. *p < 0.05 versus Gpbar1^+/+^ naïve mice, ^#^p < 0.05 versus Gpbar1^+/+^HFD-F mice. (**D**) Effects of BAR501 on mRNA expression of BAT genes. Results are the mean ± SE of 5 mice per group. *p < 0.05 versus Gpbar1^+/+^ naïve mice, ^#^p < 0.05 versus Gpbar1^+/+^ HFD-F mice. Values are normalized to β2-Microglobulin and Actin-β, the relative mRNA expression is expressed as 2^(−ΔΔCt)^ as described in Materials and Methods.

As shown in Fig. 7A, feeding mice with a HFD-F caused a slight reduction of BAT weight. In contrast, treating mice with BAR501 increased the weight of BAT and increased its thermogenic activity (P < 0.05 vs HFD-F). A mean increase of temperature of 1 °C, from 34.4 to 35.5 °C, was measured at infrared spectroscopy analysis of BAT in mice administered BAR501 in comparison to mice fed a HFD-F (Fig. 7B and C; P < 0.05). Furthermore, BAR501 increased the expression of Pgc-1α mRNA in the BAT (Fig. 7D; P < 0.05).

### BAR501 remodels bile acid synthesis

**Figure 8:**
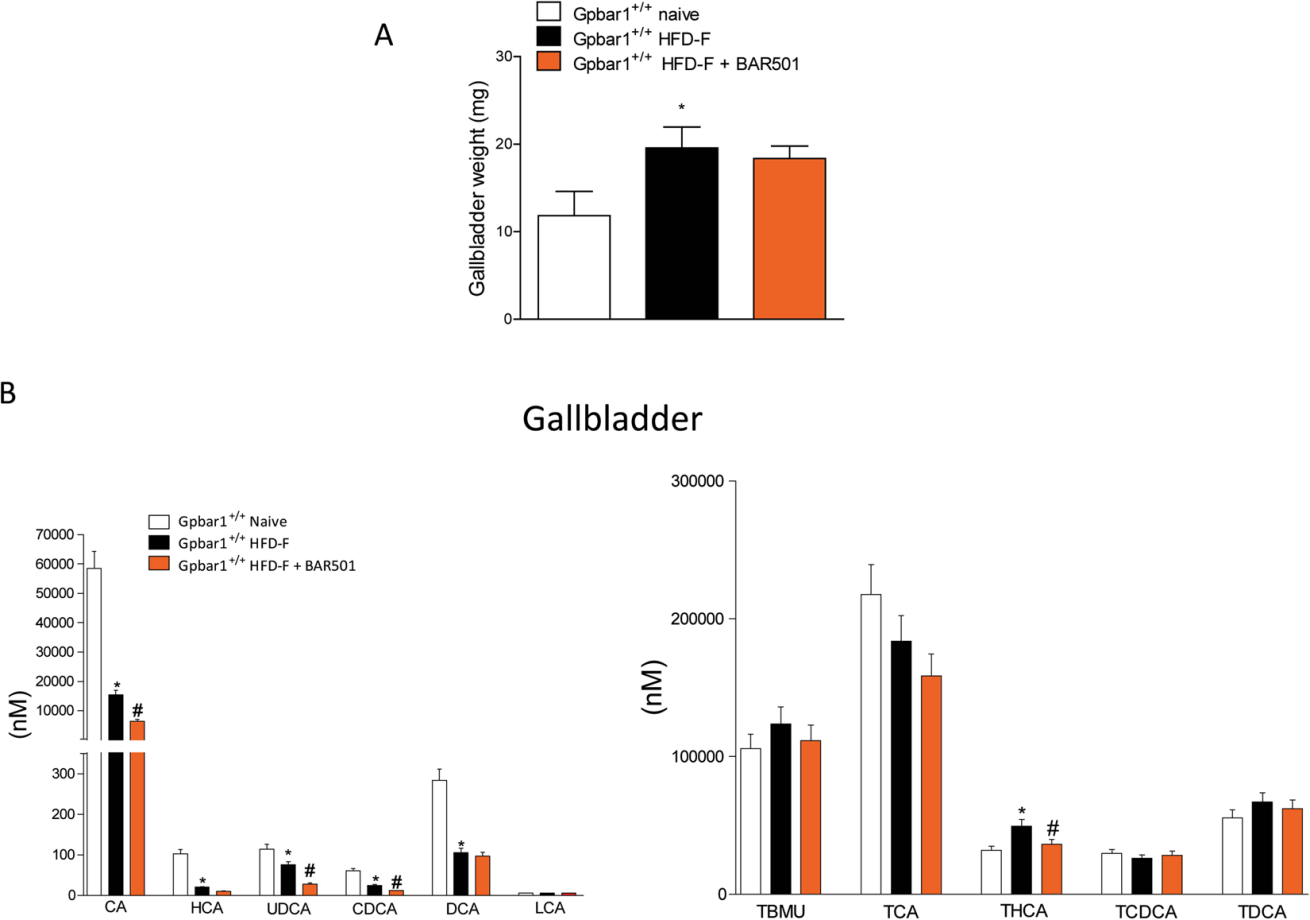
Effect of BAR501 on gallbladder volume and gallbladder bile acids composition in mice feed HFD-F. (**A**) Gallbladder weight and (**B**) bile acid species. Data are the mean ± SE of 6 mice per group. *p < 0.05 versus Gpbar1^+/+^ naïve mice, ^#^p < 0.05 versus Gpbar1^+/+^HFD-F mice.

Dosing HFD-F mice with BAR501 had no effect on gallbladder weight (Fig. 8A), and composition of bile acids as indicated by analysis of gallbladder content of free and conjugated bile acids (Fig. 8B).

### The beneficial effects exerted by BAR501 are Gpbar1-dependent

To investigate whether the effects exerted by BAR501 were due to Gpbar1 activation, Gpbar1^−/−^ mice were fed with 15 mg/day BAR501 for 4 weeks. As shown in Supplementary Figure 1, however, administration of BAR501 to Gpbar1^−/−^ mice failed to modulate insulin sensitivity as measured by OGTT and had no effects on liver histopathology. Taken together, these data indicate that BAR501 acts through a Gpbar1-mediated mechanism.

### BAR501 promotes the browning of pre-adipocytes *in vitro*

**Figure 9:**
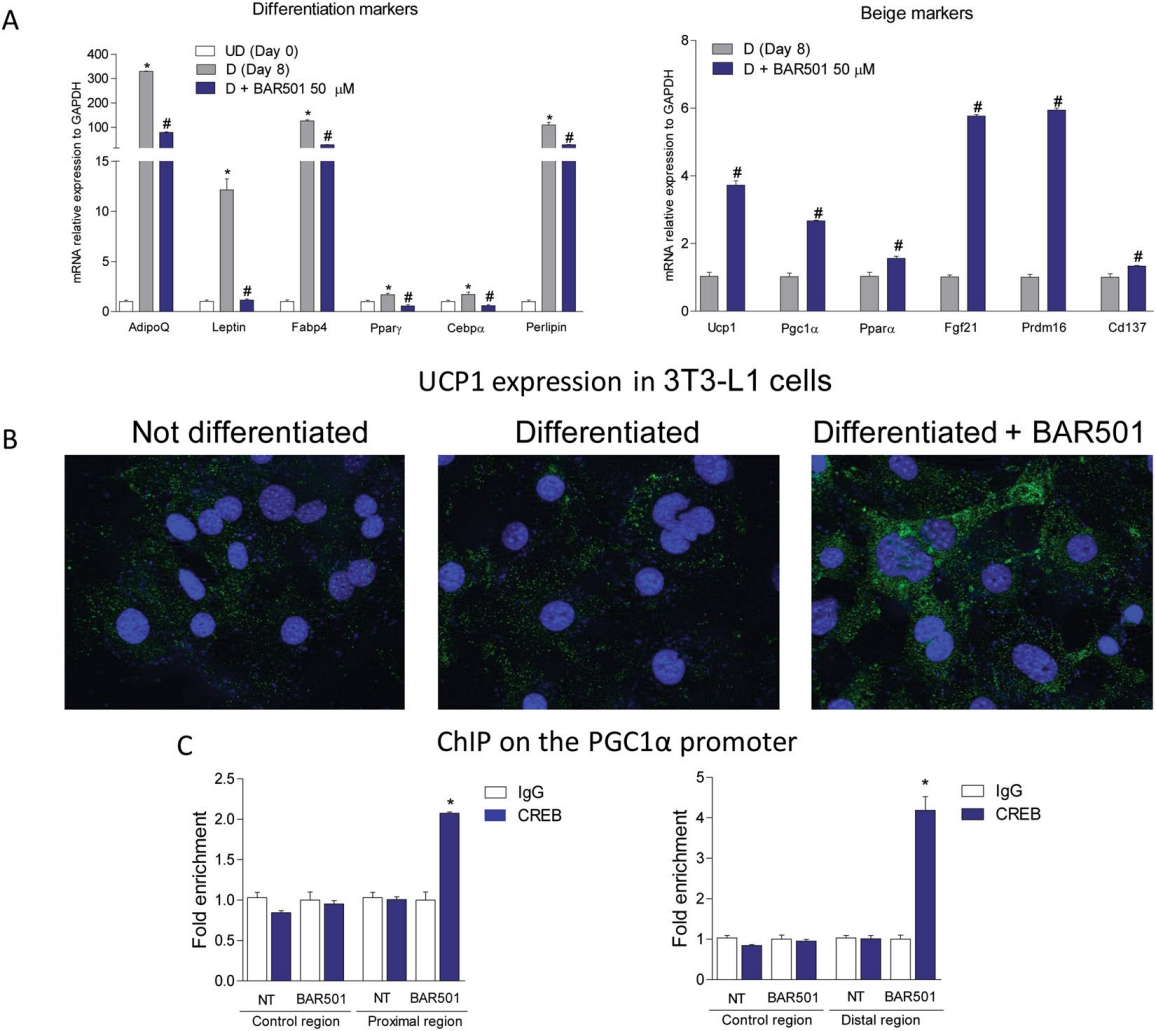
Exposure of 3T3-L1 cells to BAR501 induces the brite transdifferentiation. (**A**) 3T3-L1 were differentiated for 8 days and then exposed for 24 hours to 10 μM (Supplementary Figure 2) or 50 µM BAR501. Total RNA was extracted from cells and used to evaluate the relative mRNA expression of adipogenic marker genes and genes involved in brite differentiation. Results are the mean ± SE of 2 experiments carried out in triplicate. *p < 0.05 versus undifferentiated cells (Day 0); ^#^p < 0.05 versus differentiated cells (Day 8). Values are normalized to Gapdh, the relative mRNA expression is expressed as 2^(−ΔΔCt)^. Brite transdifferentiation was detected by a UCP1 staining on 3T3-L1 cells. (**B**) Immunofluorescence revealed that cells treated with BAR501 (50 µM), showed an enhanced Ucp1 staining compared both with undifferentiated and differentiated cells. Magnification 63x. (**C**) Chromatin immunoprecipitation with anti-pCREB antibody on 3T3-L1 cells differentiated 8 days and stimulated with 50 µM BAR501 for 18 h. Real-Time PCR was performed on Pgc-1α murine promoter (proximal, distal and control regions) to demonstrate the recruitment of the transcription factor pCREB. Precipitation with an unrelated antibody (anti-IgG) was used as negative control. Values are normalized with Fold Enrichment Method and expressed as 2^(−ΔΔCt)^. *p < 0.05 versus Negative Control (anti-IgG).

As shown in Fig. 9A, exposure of 3T3-L1 cells to differentiating mixture in presence of BAR501 (50 and 10 µM), added during the last 24 hrs of incubation, partially reversed the expression of markers of white differentiation and promoted the expression of markers of beige differentiation (Fig. 9A and Supplementary Figure 2; n = 3, *p < 0.05 versus Day 0; #p < 0.05 versus Day8). Thus culturing 3T3 pre-adipocytes with BAR501, increased expression of marker of browning differentiation including Ucp1, Pgc-1α (2–4 folds), Pparα, Fgf21 and Prdm16 (≈6 folds). Together with a reduction of adiponectin (from 300 to 90 folds increase; p < 0.01) and leptin (from ≈12 folds to no effect; p < 0.01) among other genes, these data strongly indicate that BAR501 promotes the differentiation of 3T3-L1 cells toward a beige phenotype. To confirm these results, we stained 3T3-L1 cells with anti-Ucp1 antibody. As shown in Fig. 9B, both the pre-adipocytes and differentiated 3T3 cells, present a weak staining of Ucp1. Conversely treating differentiated cells with BAR501, strongly increased the Ucp1 signal in the cytoplasm (Fig. 9B).

Since CREB induces Pgc-1α by binding to a CRE in the Pgc-1α promoter and recruiting coactivator proteins such as Cbp^6^, we performed ChIP analysis for CREB using extracts from 3T3 cells differentiated (8 days) and treated with vehicle or BAR501 (50 µM). As shown in Fig. 9C, exposure to BAR501 increased CREB binding to CRE sequence in the Pgc-1α promoter (both in the proximal and in the distal regions), indicating that Gpbar1 ligand directly activates Pgc-1α in promoter-dependent manner.

## Discussion

Gpbar1, a bile acid activated receptor, functions as a nutrient sensor activated mainly (but not exclusively) by secondary bile acids^4^. Although human and murine hepatocytes do not express Gpbar1, the receptor is highly expressed in the intestine, adipose tissues and muscles and it is thought to play a role in regulating energy expenditure^7^.

In the present study we demonstrate that Gpbar1 has an ancillary role in modulating the expression of genes involved in lipid and glucose homeostasis in the liver^13^, but impacts on bile acid synthesis. We have previously shown that bile acid pool size increases significantly in Gpbar1^−/−^ mice^13^ and this associates with a robust increase in the amount of more hydrophobic bile acids such as TCA. More recently, Donepudi *et al*.^14^ have reported that Gpbar1^−/−^ mice on a chow diet have reduced liver expression of Cyp7b1 and Cyp27a1, which are the starting points of the alternative (acidic) pathway that leads to preferential generation of CDCA, supporting a role for this receptor in the regulation of bile acid homeostasis.

In addition, we have found that Gpbar1 gene ablation resulted in several genes abnormalities in the epWAT, BAT, muscles and intestine. Along with a widespread downregulation of Pgc-1α mRNA, Gpbar1^−/−^ mice were characterized by a reduced expression of genes involved in mitochondrial energy metabolism, such as Ucp1, in the epWAT and muscles. Additionally, a reduced expression of Fgf-15 and Glp-1 mRNAs was detected in the small intestine^5^. Taken together, these data are confirmatory of a possible role of Gpbar1 in regulating energy metabolism in the adipose tissues. However, it has to be noted that Gpbar1^−/−^ mice are characterized by an increased energy expenditure being significantly more active than their congenic littermates^14^.

Consistent with these findings, feeding Gpbar1^−/−^ mice with a HFD-F did not resulted in a liver specific phenotype. Indeed, while Gpbar1^−/−^ gained weight slower than their congenic littermates, this trends attenuated with time and after 9 weeks of HFD-F there was no difference in the percent of body weight gained and severity of insulin resistance. This pattern was maintained up to the end of the study, and no difference was detected in term of liver damage as assessed by measuring steatosis, inflammatory and fibrosis scores after 18 weeks of HFD-F.

Because these data suggest that Gpbar1 gene ablation does not directly regulate liver metabolism, but is required for lipid and glucose homeostasis in adipose tissues, we have investigate whether activation of the receptor by a selective ligand could reverse NASH like feature in wild type mice fed a HFD-F. For this purpose, HFD-F mice were administered with BAR501, a non-bile acid, steroidal, Gpbar1 ligand^9^. Previous studies have shown that BAR501 is a selective Gpbar1 ligand with no activity on FXR and other bile acid activated receptors, including LXRs and VDR^9,10^. Here, we report that 9 weeks treatment with BAR501 reverses liver damage in a mouse model of NASH.

Indeed, treating mice with BAR501 reduced the expression of inflammatory markers in the liver. Of relevance, despite the fact that Gpbar1 is not expressed in liver parenchimal cells, both resident macrophages^5^ and migrated monocytes express the receptor^5,16^ and we have recently shown that activation of Gpbar1 by BAR501, shifts the monocytes phenotype toward a M2 (less inflammatory) phenotype^17^. This robust anti-inflammatory activity of BAR501 is likely to contribute to the beneficial effects observed in this study^17^.

The beneficial effects exerted by BAR501 were Gpbar1 dependent and spanned on several metabolic pathways. Thus, BAR501 increased insulin sensitivity and reshaped adipose tissues/liver lipid partitioning by causing a 50–70/% reduction of liver ballooning, inflammatory and fibrosis scores. Reduction of liver injury was linked *bonafide* to the removal of cholesterol and triglycerides from the liver and their redistribution into adipose tissues, as demonstrated by increased blood levels of cholesterol and HDL and increased weight of epWAT and weight and functionality of BAT. Treating HFD-F mice with BAR501, improved the overall WAT health and promoted the transition of epWAT adipocytes toward a beige/brown phenotype as indicated by enhanced expression of Ucp1, mRNA and protein, and Pgc-1α^18,19^. Because Ucp1 resides in the mitochondrial inner membrane and acts as a proton channel which dissipates proton motive force as heat instead of ATP production^19–21^, these findings suggest that BAR501 increased the thermogenic function of the beige/brite adipocytes.

BAT is a major site of non-shivering thermogenesis in mammals^22,23^. In adult humans, active BAT has been identified^22,23^ in the upper trunk (i.e., in cervical, supraclavicular, paravertebral, pericardial, and, to some extent, mediastinal and mesenteric areas). These BAT depots are largely composed of beige/brite cells and identified as BAT via analysis of cell morphology and expression of UCP1^22,23^. The human BAT activity is inversely associated with obesity, age, and type II diabetes, making this tissue an interesting therapeutic target^24^. Our results demonstrate that while the volume of BAT is reduced in response to feeding mice a HFD-F, its thermogenic activity increases. Treating mice with BAR501 resulted in a further increase in BAT functionality, as confirmed by the ≈30% increase in the BAT weight, and its thermogenic activity as measured by infra-red spectroscopy showed an increase of ≈1 °C in comparison with mice fed HFD-F alone. These finding associated with an increased expression of Pgc-1α and Dio2 mRNAs. Taken together, these data suggest that activation of Gpbar1 in a rodent model of NASH, protects the liver by increasing energy expenditure and functionality of BAT and promoting the browning of epWAT.

Analysis of nuclear receptors demonstrated that Gpbar1 activation by BAR501 regulates the expression of Pgc-1α in the epWAT, BAT and muscles. Pgc-1α is a master regulatory gene that binds to and co-activate several nuclear receptors and transcription factors^19^, exerting an essential role in metabolic reprogramming in response to dietary availability through coordination of the expression of genes involved in glucose, fatty acid and cholesterol homeostasis. Further on, Pgc-1α confers on WAT some of the attributes of BAT, including high mitochondrial content and the induction of Ucp-1^19^.

To investigate how Gpbar1 regulates Pgc-1α gene expression, we have used an adipocyte cell lines. The 3T3-L1 pre-adipocytes can be differentiated into mature adipocytes by exposure to a mix of inducing factors. Confirming the in *vivo* studies, we have observed that culturing pre-adipocytes with BAR501, attenuates their differentiation toward a white phenotype, while promotes the acquisition of a “beige” phenotype as indicated by increased expression of well characterized marker of browning differentiation such Ucp1, Pgc-1α, Pparα, Fgf21 and Prdm16. Further, by *in vitro* investigation, have shown that Gpbar1 activation directly regulates Pgc-1α gene expression. Indeed, the results of our ChIP experiments demonstrate that under Gpbar1 activation by BAR501, CREB is recruited to a CRE located in the Pgc-1α promoter. Previous studies have shown that Pgc-1α is induced by CREB during fasting^6^ and overexpression of Pgc-1α in CREB-deficient mice restores gluconeogenic genes expression and glucose homeostasis^6^, firmly establishing a role for CREB in regulating Pgc-1α^25^. In summary, present data strongly indicate, that secondary bile acids might regulate Pgc-1α through a Gpbar1-cAMP-CREB cascade in adipose tissues, exerting an essential role in maintaining energy homeostasis in response to fasting-feeding cycles^26^.

In conclusion, we have provided evidence that BAR501, a Gpbar1 ligand, promotes a Gpbar1-CREB dependent browning of epWAT and thermogenesis of BAT and protects the liver in a mouse model of NASH.

## Electronic supplementary material

Supplementary information
