# Supplementary material for: Gpbar1 agonism promotes a Pgc-1α-dependent browning of white adipose tissue and energy expenditure and reverses diet-induced steatohepatitis in mice
Source: Sci Rep. 2017 Oct 20;7:13689. doi: 10.1038/s41598-017-13102-y (PMC5651899; doi:10.1038/s41598-017-13102-y)
Supplement: Supplementary file 1 — Supplementary information [file 41598_2017_13102_MOESM1_ESM.pdf]

Gpbar1 agonism promotes a Pgc-1α-dependent browning of white adipose tissue and energy expenditure and reverses diet-induced steatohepatitis in mice

Adriana Carino[1#], Sabrina Cipriani[2#], Silvia Marchianò[1], Michele Biagioli[1], Paolo Scarpelli[5]
Angela Zampella[3], Maria Chiara Monti[4], and Stefano Fiorucci[1*]

[#]contributed equally to this study

[1] University of Perugia, Department Surgical and Biomedical Sciences, Perugia, Italy
[2] University of Perugia, Department of Medicine, Perugia, Italy
[3] University of Naples Federico II, Department of Pharmacy, Naples, Italy
[4] University of Salerno, Department of Pharmacy, Fisciano, Salerno, Italy
[5] University of Perugia, Department of Experimental Medicine, Perugia, Italy

Corresponding author:
Stefano Fiorucci, M.D.
University of Perugia Medical School
Department Surgical and Biomedical Sciences
Tel. +390755858120
email: stefano.fiorucci @unipg.it

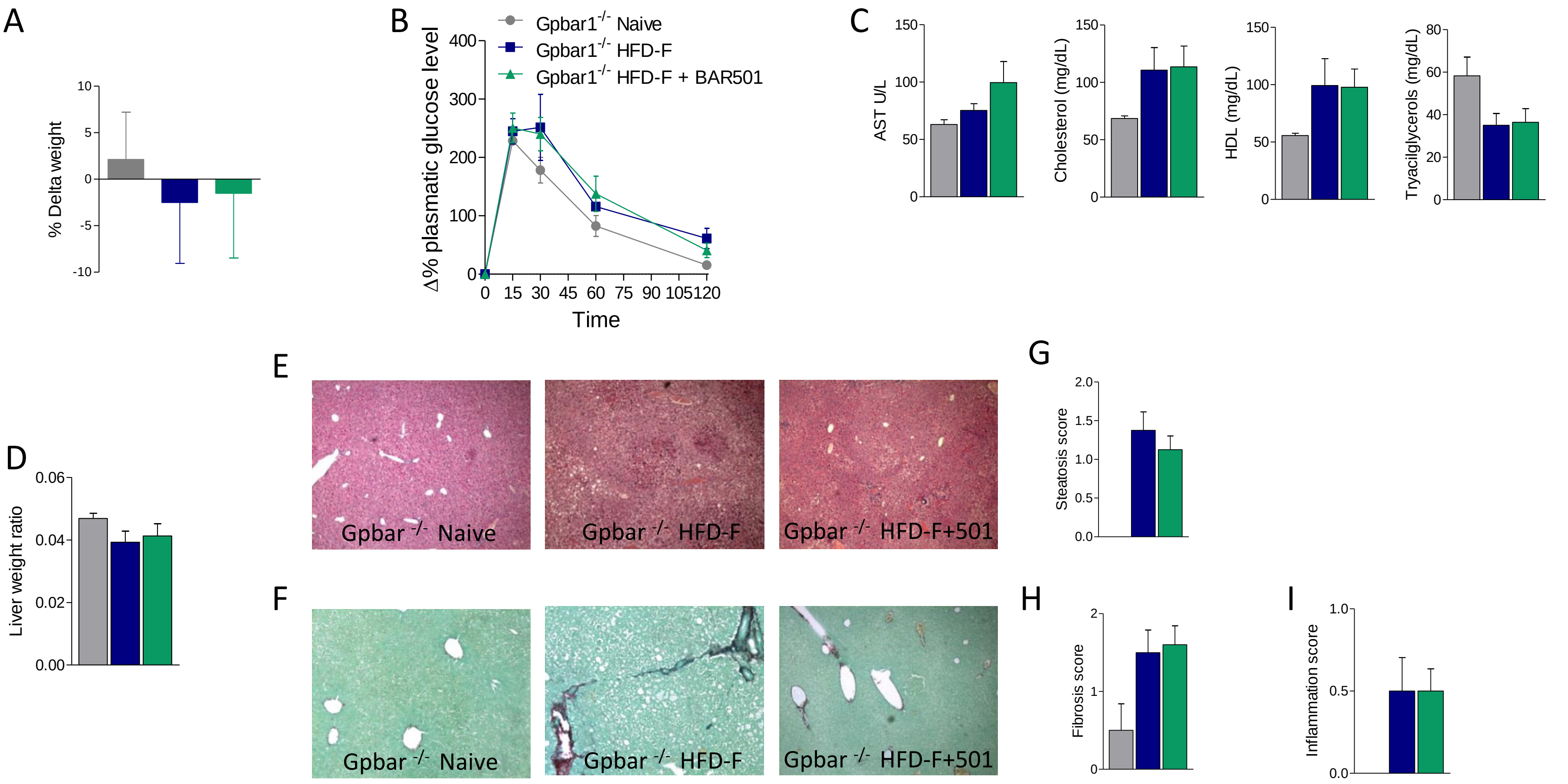

Supplementary Figure 1. Mice were fed a HFD (High fat diet) and fructose for 18 weeks. BAR501 was administered at the dose of 15 mg/kg/day starting on day 114. The data shown are: (A) body weight (% delta weight); (B) glycemic response to oral glucose tolerance test (OGTT) (after 18 weeks of HFD); (C) plasma levels of AST, cholesterol, HDL and triacylglycerols measured at the end of the study; (D) Liver weight ratio; (E) Hematoxylin and eosin (H&E) staining on mice liver tissues showing severe steatosis in mice feed a HFD for 19 weeks; these changes were not attenuated by treating the HFD mice with BAR501. (F) Sirius red staining of liver sections. Panels G-I. Impact of BAR501 on (G) steatosis (Steatosis score), (H) Inflammation score and (I) Fibrosis score. The data are mean ± SE of 9 mice.

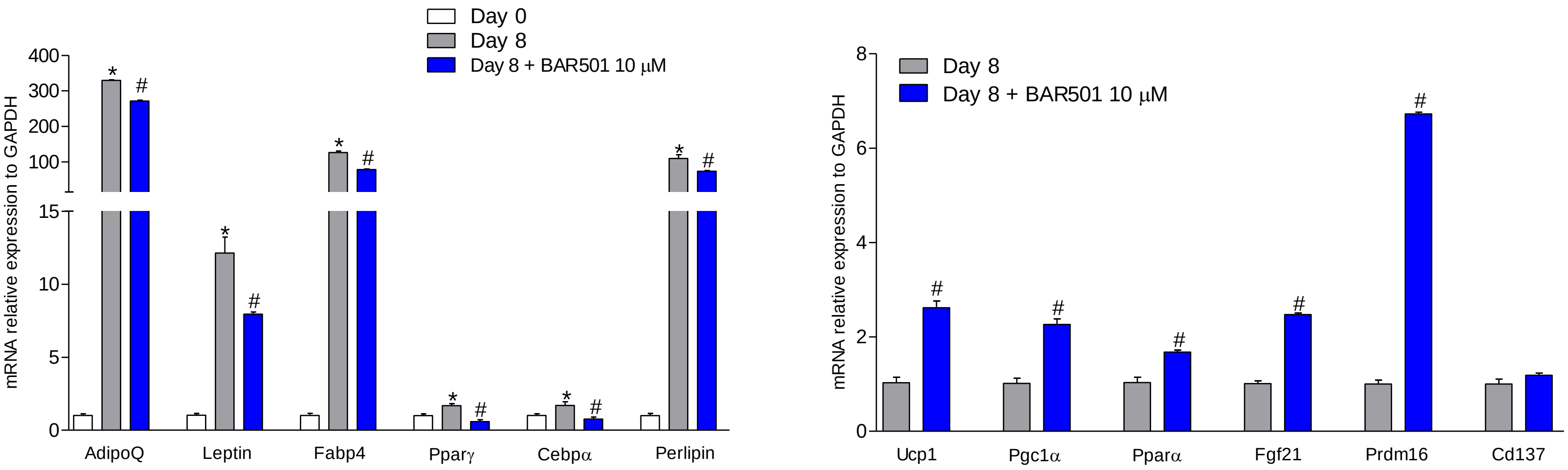

Supplementary Figure 2. 3T3-L1 were differentiated for 8 days and then exposed for 24 hours to 10 μM BAR501. Total RNA was extracted from cells and used to evaluate the relative mRNA expression of adipogenic marker genes and genes involved in brite differentiation. Results are the mean ± SE of 2 experiments carried out in triplicate. *$p<0.05$ versus undifferentiated cells (Day 0); # $p<0.05$ versus differentiated cells (Day 8). Values are normalized to GAPDH, the relative mRNA expression is expressed as $2^{(-\Delta\Delta Ct)}$.
